# Limited predictive value of traditional comorbidities for readmission in acute decompensated heart failure

**DOI:** 10.1371/journal.pone.0329829

**Published:** 2025-08-06

**Authors:** Gil Marcus, Antoinette Monayer, Gil Moravsky, Shmuel Fuchs, Avishay Grupper, Eran Kalmanovich, Sa’ar Minha

**Affiliations:** 1 Department of Cardiology, Shamir Medical Center, Be’er Ya’akov, Israel; 2 Gray Faculty of Medical & Health Sciences, Tel-Aviv University, Tel-Aviv, Israel; Universita degli Studi di Napoli Federico II, ITALY

## Abstract

**Background:**

Common comorbidities in heart failure (HF), including chronic kidney disease (CKD), diabetes mellitus (DM), ischemic heart disease (IHD), and atrial fibrillation, are frequently presumed to predict hospital readmission. However, recent studies have challenged their predictive strength, raising questions about their clinical utility for risk stratification.

**Methods:**

We conducted a retrospective cohort study of 7,652 patients admitted with acute decompensated heart failure (ADHF) at a tertiary center between 2007 and 2017. Associations between comorbidities and readmission at 30 and 100 days were assessed using Fine-Gray competing risk models, with death as a competing event. Subdistribution hazard ratios (sHRs) were reported. Model performance was evaluated using receiver operating characteristic (ROC) analysis and area under the curve (AUC) values, assessing individual comorbidities and incremental combinations. All comorbidities were included irrespective of univariable significance, based on clinical relevance.

**Results:**

Several comorbidities were significantly associated with readmission, including CKD (sHR 1.16–1.23), DM (sHR 1.18–1.27), IHD (sHR 1.10–1.15), and anemia (sHR 1.11). However, predictive power was poor. For 30-day readmission, AUC values ranged from 0.516 (COPD) to 0.529 (CKD), with a maximal AUC of 0.555 when combining the four strongest predictors. For 100-day readmission, AUC values ranged from 0.528 (DM) to 0.545 (CKD), with a maximal combined AUC of 0.593.

**Conclusions:**

Despite consistent statistical associations, common comorbidities perform poorly as predictive tools for identifying individual patients at risk of HF readmission. These findings highlight the need for more robust risk models integrating dynamic clinical, laboratory, and patient-centered factors.

## Introduction

Heart failure (HF) is a prevalent clinical syndrome associated with high rates of hospitalization and mortality [1–3]. HF readmissions represent a substantial burden on healthcare resources and are associated with significant costs and worse patient outcomes [4–6]. Reducing readmissions has therefore been recognized as a national priority in the United States, exemplified by initiatives such as the Hospital Readmissions Reduction Program (HRRP) [7]. This has prompted research efforts to identify predictors of readmission that could inform pre-discharge checklists or post-discharge care strategies aimed at lowering readmission rates. Common comorbidities in HF patients, such as atrial fibrillation (AF), chronic kidney disease (CKD), and chronic obstructive pulmonary disease (COPD), have been consistently associated with an elevated risk of adverse outcomes, including hospital readmissions [8–10].

However, a recent study by Scholten et al. challenged the importance of these comorbidities as predictors of readmission in HF patients. Their study found that most commonly recognized comorbidities were not significantly associated with increased 100-day readmission risk, and even for comorbidities that did show significant associations, such as AF, CKD, and COPD, their predictive power was limited, as demonstrated by low area under the curve (AUC) values in receiver operating characteristic (ROC) analyses [11]. These findings raise important questions about the clinical value of traditionally recognized predictors of HF readmission. Furthermore, since most prior studies focused exclusively on the conventional outcome measure of 30-day readmissions, it remains unclear whether the limited predictive ability demonstrated by Scholten et al. at 100 days is similarly evident at this earlier standard time-point.

To address these questions, we elected to reassess the association and predictive power (evaluated by ROC-AUC analysis) of traditional comorbidities linked to HF readmission at 30 and 100 days, in a large cohort of patients admitted with acute decompensated HF (ADHF) and followed for an extended period.

## Methods

### Study design and population

This retrospective cohort study included adult patients admitted with a primary diagnosis of acute decompensated heart failure (ADHF) at Shamir Medical Center, Be’er Ya’akov, Israel, between January 1, 2007, and December 31, 2017. Eligible patients were those aged 18 years or older, diagnosed with ADHF at admission and at discharge based on ICD-9 codes 428.xx, 429.xx, and 514. Patients who died during the index admission were excluded from the analysis.

Baseline demographic and clinical characteristics, including age, sex, and comorbidities, were obtained from the hospital’s electronic medical record system. Comorbidities assessed included AF, COPD, diabetes mellitus (DM), ischemic heart disease (IHD), peripheral vascular disease (PVD), renal failure (RF), and anemia at admission. The primary outcomes were all-cause hospital readmission within 30 days and within 100 days following discharge. Mortality data were obtained from the national registry of the Israeli Ministry of Interior Affairs and were incorporated as a competing risk in the analysis.

The study was approved by the institutional review board (Helsinki Subcommittee) of Shamir Medical Center under IRB number ASF-0063-18, with initial approval granted on April 23, 2018 and the most recent extension approved on March 25, 2025. Owing to the retrospective design and use of anonymized data for reporting, the requirement for informed consent was waived. Data for this study were first accessed for research purposes on March 13, 2025. During data collection and analysis, the dataset included identifying information. However, access to the raw data was restricted to the physician investigator with institutional authorization. All reported results, including tables and figures, are based solely on aggregated data and contain no small cells or other features that could permit identification of individual patients.

This study was reported in accordance with the Strengthening the Reporting of Observational Studies in Epidemiology (STROBE) guidelines for cohort studies [12].

### Handling of missing data

Anemia status at admission had 12% missing data, while all covariates used in the analysis were fully complete. Because the present study used Fine-Gray competing risks models (see below), which require complete covariate data and cannot accommodate missing values, missing anemia values were imputed using predictive mean matching (PMM). The imputation model included all other covariates used in the analysis (age, sex, AF, COPD, DM, IHD, PVD, RF) and excluded outcome variables to avoid introducing bias. Five iterations were performed within the imputation process to allow the model to stabilize the imputed values. To examine the impact of the imputation assumptions on the final results, a sensitivity analysis was conducted by comparing ROC performance before and after imputation for both 30-day and 100-day readmission. The AUC values differed by less than 0.005, indicating minimal influence of the imputation strategy. The completed imputed dataset was then used for all subsequent analyses.

### Statistical analysis

Age is presented as mean ± standard deviation (SD), and categorical variables are presented as n (%). Group comparisons were performed using Student’s t-test for age and Fisher’s exact test for categorical variables.

The association between comorbidities and readmission risk was assessed using Fine-Gray competing risk proportional hazards models, with hospital readmission as the event of interest and death within the respective follow-up period as a competing risk. Fine-Gray models estimate the hazard of the event of interest by modeling its cumulative incidence over time while accounting for competing events; specifically, patients who experience the competing event (such as death) are retained in the risk set but are no longer able to have the primary event, which avoids overestimating the event probability. Because these individuals remain in the risk set but cannot experience the event, the calculated distribution is referred to as a subdistribution hazard, rather than a cause-specific hazard [13]. Separate models were constructed for 30-day and 100-day readmission outcomes. Subdistribution hazard ratios (sHRs) with 95% confidence intervals (CIs) were reported for each comorbidity. All covariates were included in the multivariable Fine-Gray competing risks models irrespective of their univariable significance, based on their literature-established clinical relevance. This included anemia, [14] CKD, [15] DM, [16] IHD, [17] AF, [18] COPD, [19] and PVD [20]—each of which has been consistently associated with adverse outcomes in heart failure populations.

Model performance was then evaluated using receiver operating characteristic (ROC) curve analysis and the area under the curve (AUC). In contrast to the all-covariate approach used in the multivariable models, here only the comorbidities that remained independently significant after the multivariable models were included in the ROC analyses. ROC curves were constructed separately for the 30-day and 100-day readmission models. AUC values were interpreted as follows: 0.5 indicating no discrimination, 0.6–0.7 poor, 0.7–0.8 acceptable, 0.8–0.9 excellent, and >0.9 outstanding discrimination.

All statistical analyses were conducted using R version 4.2.0 (R Core Team, 2022) and Python version 3.10. Two-tailed p-values <0.05 were considered statistically significant.

## Results

### Patient characteristics

The study cohort included 7,652 patients admitted with ADHF. Of these, 1,670 patients (21.8%) were readmitted within 30 days of discharge, and 2,959 patients (38.7%) were readmitted within 100 days.

Baseline demographic characteristics and comorbidity distributions stratified by 30-day and 100-day readmission status are presented in Table 1. Age did not differ significantly between readmitted and non-readmitted patients at either timepoint, with an overall mean of 76.0 ± 12.0 years. Sex distribution was also similar at 30 days (47.4% vs. 49.8% female, p = 0.078) but differed at 100 days, with a lower proportion of females among those readmitted (47.8% vs. 50.2%, p = 0.035). Several comorbidities were consistently more prevalent among readmitted patients. Notably, CKD was present in 38.1% of patients readmitted within 30 days compared to 32.3% of those not readmitted (p < 0.001), and in 39.1% vs. 30.1% at 100 days (p < 0.001). Anemia showed a similarly strong pattern (71.0% vs. 65.0% at 30 days, p < 0.001; 71.2% vs. 63.3% at 100 days, p < 0.001). Other comorbidities, including IHD, AF, DM, and COPD, were also significantly more prevalent among readmitted patients at both timepoints. PVD was not associated with 30-day readmission but showed higher prevalence at 100 days (8.2% vs. 6.6%, p = 0.009).

**Table 1:** Comorbidities, Age, and Sex Stratified by 30-Day and 100-Day Readmission Status.

	Readmitted within 30 days		p- value	Readmitted within 100 days		p-value
	Yes (N = 1670)	No (N = 5955)		Yes (N = 2959)	No (N = 4666)	
Female sex, n (%)	791 (47.4)	2966 (49.8)	0.078	1413 (47.8)	2344 (50.2)	0.035
Age, years, mean±SD	76.0 ± 12.0	75.6 ± 12.1	0.193	75.8 ± 11.9	75.6 ± 12.2	0.416
Traditional readmission perdictors – n (%)						
Ischemic heart disease	743 (44.5)	2384 (40.0)	0.001	1340 (45.3)	1787 (38.3)	<0.001
Chronic kidney disease	636 (38.1)	1925 (32.3)	<0.001	1156 (39.1)	1405 (30.1)	<0.001
Atrial fibrillation	590 (35.3)	1819 (30.5)	<0.001	1059 (35.8)	1350 (28.9)	<0.001
Diabetes mellitus	887 (53.1)	2999 (50.4)	0.047	1608 (54.3)	2278 (48.8)	<0.001
Chronic obsrtuctive pulmonary disease	310 (18.6)	914 (15.3)	0.002	579 (19.6)	645 (13.8)	<0.001
Peripheral vascular disease	134 (8.0)	420 (7.1)	0.177	244 (8.2)	310 (6.6)	0.009
Anemia	1027 (71.0)	3426 (65.0)	<0.001	1829 (71.2)	2624 (63.3)	<0.001

Values are presented as n (%) for categorical variables and mean ± SD for continuous variables. Percentages represent column percentages. P-values reflect comparisons between readmitted and non-readmitted groups at each timepoint.

### Comorbidity overlap

**Fig 1:**
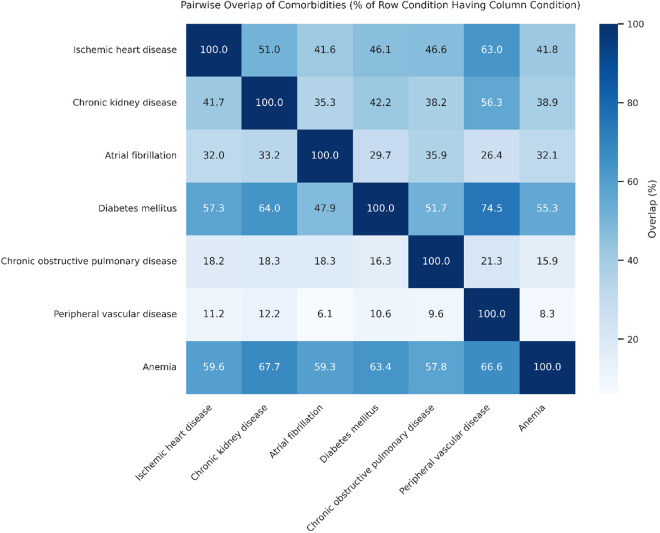
Overlap Between Comorbidities in the Study Cohort. Heatmap illustrating the percentage of patients with each comorbidity (rows) who also had a second comorbidity (columns). Values represent row-wise percentages; that is, each cell indicates the proportion of patients with the row condition who also had the column condition. A prominent vascular cluster was observed among diabetes mellitus, ischemic heart disease, chronic kidney disease, and peripheral vascular disease. Anemia demonstrated broad overlap across most conditions, while chronic obstructive pulmonary disease (COPD) showed relatively low overlap with others, suggesting relative isolation.

Beyond individual comorbidity characteristics, extensive overlap was observed between conditions, as illustrated in Fig 1. A prominent cluster was evident among vascular-related comorbidities, with DM co-occurring in 74.5% of patients with PVD, 64.0% of those with CKD, and 57.3% of those with IHD. Anemia demonstrated broad overlap across all comorbidities, present in 67.7% of patients with CKD, 66.6% with PVD, and 63.4% with DM. In contrast, COPD showed consistently low overlap with other conditions, ranging from 9.6% to 21.3%, indicating relative isolation from the vascular cluster. Notably, overlaps were often asymmetric—for example, while 64.0% of patients with CKD also had DM, only 42.2% of DM had CKD. Similarly, 59.6% of patients with IHD had anemia, compared to just 41.8% of those with anemia having IHD. The strongest asymmetry was observed between PVD and IHD: 63.0% of patients with PVD had IHD, while only 11.2% of IHD patients had PVD.

### Association *between* comorbidities and readmission risk

Subdistribution hazard ratios (sHRs) with 95% confidence intervals (CIs) for 30-day and 100-day readmissions are presented in Table 2. At 30 days, CKD (sHR 1.16, 95% CI 1.04–1.28, p = 0.007), AF (sHR 1.19, 95% CI 1.08–1.32, p < 0.001), COPD (sHR 1.20, 95% CI 1.06–1.35, p = 0.005), and anemia (sHR 1.20, 95% CI 1.07–1.36, p = 0.002) were significantly associated with higher readmission risk. IHD showed a borderline association (sHR 1.10, 95% CI 1.00–1.22, p = 0.055), while DM, PVD, female sex, and age were not significantly associated with 30-day readmission.

**Table 2:** Fine-Gray Competing Risks Models for 30-Day and 100-Day Readmissions.

	30-day readmission	p-value	100-day readmission	p-value
	sHR (95% CI)		sHR (95% CI)	
Female sex	0.9605 (0.8683–1.062)	0.43	0.9911 (0.9189–1.069)	0.82
Age	1.001 (0.997–1.006)	0.54	0.9992 (0.9959–1.002)	0.62
Ischemic heart disease	1.102 (0.998–1.218)	0.055	1.154 (1.071–1.243)	<0.001
Chronic kidney disease	1.155 (1.041–1.282)	0.007	1.225 (1.133–1.324)	<0.001
Atrial fibrillation	1.191 (1.076–1.318)	<0.001	1.248 (1.157–1.347)	<0.001
Diabetes mellitus	1.041 (0.941–1.152)	0.43	1.088 (1.008–1.174)	0.03
Chronic obsrtuctive pulmonary disease	1.196 (1.057–1.353)	0.005	1.311 (1.197–1.435)	<0.001
Peripheral vascular disease	1.021 (0.854–1.222)	0.82	1.029 (0.9018–1.174)	0.67
Anemia	1.204 (1.07–1.355)	0.002	1.235 (1.131–1.349)	<0.001

Subdistribution hazard ratios (sHR) with 95% confidence intervals (CIs) and corresponding p-values are reported for each variable, at each timepoint.

At 100 days, the pattern was similar but with additional significant associations. CKD (sHR 1.23, 95% CI 1.13–1.32, p < 0.001), AF (sHR 1.25, 95% CI 1.16–1.35, p < 0.001), COPD (sHR 1.31, 95% CI 1.20–1.44, p < 0.001), anemia (sHR 1.24, 95% CI 1.13–1.35, p < 0.001), IHD (sHR 1.15, 95% CI 1.07–1.24, p < 0.001), and DM (sHR 1.09, 95% CI 1.01–1.17, p = 0.030) were significantly associated with increased readmission risk at this timepoint. PVD, female sex, and age were not significantly associated with 100-day readmission.

### Predictive power of comorbidities for readmission

**Fig 2:**
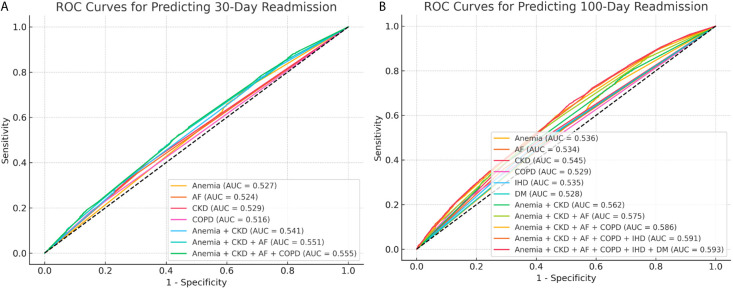
Receiver Operating Characteristic (ROC) Curves for Readmission Prediction by Comorbidities. AUC values are shown for individual comorbidities that remained independently significant in the multivariable Fine-Gray models, as well as for their incremental combinations. For 30-day readmission (Figure a), the maximal AUC achieved was 0.555, combining the four significant comorbidities: CKD, COPD, DM, anemia. For 100-day readmission (Figure b), the maximal AUC achieved was 0.593, combining the six significant comorbidities: CKD, DM, anemia, ischemic heart disease, peripheral vascular disease, and COPD.

The ROC AUC was assessed for each comorbidity that remained independently significant in the Fine-Gray multivariable competing risks models. For 30-day readmission (Fig 2a), AUC values for individual comorbidities ranged from 0.516 (lowest, for COPD) to 0.529 (highest, for CKD). Incremental combinations of comorbidities were then tested, starting with the two strongest predictors—anemia and CKD—adding DM as a third, and finally IHD as the fourth. The maximal AUC achieved for 30-day readmission, with all four comorbidities combined, was 0.555.

For 100-day readmission (Fig 2b), a similar pattern was observed. AUC values for the individual six comorbidities that were found to be significant in this timeframe, ranged from 0.528 (lowest) for DM, to 0.545 (highest) for CKD. The maximal AUC achieved for 100-day readmission, with all six comorbidities combined, was 0.593.

## Discussion

In this study, we demonstrated that despite well-established associations between common comorbidities and HF readmission risk, their ability to predict which patients will be readmitted remained poor. At both 30 and 100 days after admission for ADHF, the discriminative power of these comorbidities, assessed by AUC analysis, did not exceed 0.6. These findings challenge the clinical utility of these comorbidities as effective tools for readmission risk stratification.

Our results are consistent with the findings of Scholten et al., who similarly reported low predictive power of common comorbidities for cardiovascular readmissions within 100 days among HF patients [11]. Their analysis, like ours, identified statistically significant associations for several comorbid conditions, yet these translated into weak discrimination when assessed by ROC-AUC. In their study, the maximal AUC achieved was 0.63 (95% CI 0.61–0.64) using logistic regression and 0.62 (95% CI 0.60–0.64) using Rasch analysis, both reflecting poor predictive performance. Other studies evaluating predictive models for HF readmission using comorbidities as input variables have reported similarly limited discriminative power, with AUC values generally ranging from 0.60 to 0.65 [9,10]. These findings align with our analysis, in which maximal AUC values were 0.555 for 30-day readmission and 0.593 for 100-day readmission, slightly lower but broadly comparable. The modest differences between studies may reflect variations in outcome definitions—such as cardiovascular-specific versus all-cause readmissions—as well as differences in modeling approaches, including our use of Fine-Gray competing risks regression to account for death as a competing event. Of note, the consistently poor discriminative performance across these varying methodologies and outcome definitions reinforces the conclusion that comorbidity profiles alone, regardless of analytic strategy, offer limited utility for predicting HF readmissions. The present study further supports this conclusion by demonstrating that the limited discriminative ability is not confined to the 100-day time frame but is already evident at the conventional 30-day readmission endpoint. Moreover, by assessing both individual comorbidities and their incremental combinations, our findings specifically tested whether additive modeling of these variables could improve risk stratification—an approach not the primary focus of previous studies—and found no substantial improvement.

While direct evaluations of predictive power remain limited, numerous observational studies and meta-analyses have consistently reported strong associations between comorbidities such as CKD, DM, IHD, and AF and adverse HF outcomes, including hospital readmissions [8,21,22]. However, as we showed, these associations, although statistically robust, do not necessarily imply strong predictive power. Several factors may contribute to this phenomenon. One is that these comorbidities are so prevalent in HF populations that they fail to meaningfully separate high- from low-risk individuals. When nearly half of all patients have a condition like DM or CKD, it raises average risk, but leaves little discriminatory signal between those who will and won’t be readmitted. Another factor is that these comorbidities reflect baseline vulnerability, but not the proximate causes of readmission, which often result from acute, dynamic events such as infections, arrhythmias, or lapses in adherence [23,24]. As a result, comorbidities may explain who is at risk for worse overall outcomes, but fail to identify who will cross the threshold into a specific readmission event within a defined timeframe.

Aside from these clinical explanations, our findings highlight a fundamental distinction between regression modeling and prediction analysis. Regression models test whether a variable shifts average risk across a population, but predictive discrimination depends on how well it separates individual outcomes. Even when mean risk differs significantly between groups, wide within-group variance can cause heavy overlap, limiting classification accuracy. Importantly, this within-group variance is not reflected by the 95% confidence interval (CI) typically reported in regression models, which describes the precision of the group mean estimate, not the spread of individual risks around that mean. Thus, a narrow 95% CI may coexist with wide individual-level variance that overlaps with risks in the comparator group, limiting discrimination and lowering predicting power [25,26]. Our study underscores the importance of reporting predictive metrics, not just regression outputs, when evaluating potential risk markers.

The selection of the 30-day and 100-day timeframes in the present analysis warrants consideration. While the 30-day period remains the standard benchmark in HF readmission research and policy, its exclusive use has been questioned. Magaña et al. introduced the concept of phased post-discharge vulnerability, identifying and describing a triphasic structure of risk following HF hospitalization: early (0–30 days), intermediate (31–90 days), and late (91–180 days) phases comorbidities in HF. In the current study, for a second timeframe beyond the early 30-day timeframe, we chose the 100-day timeframe as a pragmatic balance, aligning with the timeframe Scholten et. al chose, and aiming to capture both the intermediate and late phases within a single analysis window. Although repeating the analysis separately for these three phases might offer further granularity, we considered this beyond the scope of our study, which focused on evaluating the overall predictive power of comorbidities rather than the temporal evolution of readmission risk. The similarly poor predictive performance observed at both 30 and 100 days suggests that the limitations of comorbidity-based stratification are not restricted to a specific follow-up duration.

In addition to evaluating predictive performance, our study also identified comorbidity clustering patterns that may help illustrate the biological heterogeneity of HF. Notably, the relative isolation of COPD from vascular comorbidities such as DM, IHD, and CKD suggests that COPD may contribute to HF through distinct mechanisms. While vascular comorbidities are predominantly driven by atherosclerosis, endothelial dysfunction, and metabolic dysregulation, [27,28]. COPD is more closely associated with chronic hypoxia, pulmonary vascular remodeling, and right ventricular strain—features that may give rise to a different form of cardiac stress within the HF spectrum [29,30]. These contrasting pathways may help explain the looser clustering of COPD with vascular conditions in our cohort, despite shared systemic risk factors such as smoking and inflammation. Conversely, the broad overlap of anemia across comorbidity groups likely reflects its role as a nonspecific marker of overall disease burden, potentially driven by chronic inflammation, nutritional deficiencies, or renal impairment [31,32]. These structural patterns strengthen the notion that considering biological context and phenotypic clustering—rather than simple comorbidity counts—may be necessary to advance prognostic modeling and care stratification in HF. It is also worth noting that advances in heart failure pharmacotherapy may influence the downstream effects of comorbidities. For instance, sacubitril/valsartan has been associated with improvements in physical frailty among patients listed for heart transplantation, highlighting the potential of contemporary treatments to mitigate the clinical impact of baseline vulnerability [33]. Such developments further complicate the interpretation of static comorbidity profiles, emphasizing the need for risk models that reflect the evolving therapeutic landscape.

Our findings, together with those of Scholten et al., bring into sharp focus a critical question: should we abandon comorbidity-based risk models and look for other, perhaps more dynamic and individualized alternatives? Several recent modeling approaches suggest alternative or complementary paths. Frailty-based models, particularly those using multidimensional constructs encompassing cognitive, social, and physical domains, have shown stronger predictive value than traditional comorbidity counts [34–36]. Machine-learning tools using administrative and clinical data—such as eXtreme Gradient Boosting (XGBoost)—have reported improved accuracy in predicting HF readmissions, with AUCs reaching 0.763 [37]. Additional approaches have explored other predictors such as natriuretic peptides, [38] functional status metrics, [39] or medication adherence [40] as dynamic indicators of short-term vulnerability. While these tools each have limitations—ranging from complexity to generalizability—they reflect a clear shift toward multidimensional, patient-centered models. In this context, comorbidities may still serve a useful role, but likely as components of integrated frameworks rather than standalone predictors.

This study has several limitations. First, its retrospective design and reliance on administrative coding may have led to misclassification of comorbidities or outcomes. Second, the analysis was conducted at a single center, which may limit generalizability to other populations or healthcare settings. Third, we focused on comorbidity profiles and did not include additional variables such as laboratory data, functional measures, medication adherence, or social determinants of health—all of which may contribute meaningfully to readmission risk and are increasingly incorporated into contemporary risk prediction models to capture the dynamic and multidimensional nature of patient vulnerability. Fourth, ejection fraction (EF) phenotyping (e.g., HF with reduced vs. preserved EF) was not included in the analysis because EF data were available for only 31% of patients—insufficient for inclusion in the Fine-Gray competing risk model used in this study and too limited to support even univariable exploration of comorbidity patterns by phenotype. Finally, although multiple imputations were applied to address missing data for anemia status, residual confounding from unmeasured or imperfectly measured variables cannot be excluded.

In conclusion, while traditional comorbidities in patients with ADHF remain independently associated with increased readmission risk, their overall predictive value is limited, even when combined. These findings underscore the limitations of comorbidity-based risk models when used in isolation and highlight the need for more comprehensive predictive strategies that incorporate dynamic clinical, behavioral, and physiologic dimensions.
